# Brief educational interventions to improve performance on novel quality metrics in ambulatory settings in Kenya: A multi-site pre-post effectiveness trial

**DOI:** 10.1371/journal.pone.0174566

**Published:** 2017-04-14

**Authors:** Robert Ryan Korom, Stephanie Onguka, Peter Halestrap, Maureen McAlhaney, Mary Adam

**Affiliations:** 1 Penda Health, Nairobi, Kenya; 2 Department of Medicine, Massachusetts General Hospital, Harvard University, Boston, MA, United States of America; 3 Kabarak University, Kabarak, Kenya; 4 AIC-Kijabe Hospital, Kijabe, Kenya; Emory University Department of Medicine, UNITED STATES

## Abstract

**Background:**

The quality of primary care delivered in resource-limited settings is low. While some progress has been made using educational interventions, it is not yet clear how to sustainably improve care for common acute illnesses in the outpatient setting. Management of urinary tract infection is particularly important in resource-limited settings, where it is commonly diagnosed and associated with high levels of antimicrobial resistance. We describe an educational programme targeting non-physician health care providers and its effects on various clinical quality metrics for urinary tract infection.

**Methods:**

We used a series of educational interventions including 1) formal introduction of a clinical practice guideline, 2) peer-to-peer chart review, and 3) peer-reviewed literature describing local antimicrobial resistance patterns. Interventions were conducted for clinical officers (N = 24) at two outpatient centers near Nairobi, Kenya over a one-year period. The medical records of 474 patients with urinary tract infections were scored on five clinical quality metrics, with the primary outcome being the proportion of cases in which the guideline-recommended antibiotic was prescribed. The results at baseline and following each intervention were compared using chi-squared tests and unpaired two-tailed T-tests for significance. Logistic regression analysis was used to assess for possible confounders.

**Findings:**

Clinician adherence to the guideline-recommended antibiotic improved significantly during the study period, from 19% at baseline to 68% following all interventions (Χ^2^ = 150.7, p < 0.001). The secondary outcome of composite quality score also improved significantly from an average of 2.16 to 3.00 on a five-point scale (t = 6.58, p < 0.001). Interventions had different effects at different clinical sites; the primary outcome of appropriate antibiotic prescription was met 83% of the time at Penda Health, and 50% of the time at AICKH, possibly reflecting differences in onboarding and management of clinical officers. Logistic regression analysis showed that intervention stage and clinical site were independent predictors of the primary outcome (p < 0.0001), while all other features, including provider and patient age, were not significant at a conservative threshold of p < 0.05.

**Conclusion:**

This study shows that brief educational interventions can dramatically improve the quality of care for routine acute illnesses in the outpatient setting. Measurement of quality metrics allows for further targeting of educational interventions depending on the needs of the providers and the community. Further study is needed to expand routine measurement of quality metrics and to identify the interventions that are most effective in improving quality of care.

## Introduction

There is increasing recognition that low- and middle-income countries (LMICs) must not only improve access to health care services, but also the quality of those services in order to meet the needs of their populations [1]. Recent studies have demonstrated that healthcare provider adherence to well-established clinical guidelines is poor across both the public and private sectors in numerous LMICs [2, 3, 4]. Suboptimal quality has been documented in both inpatient and outpatient settings, and has been attributed to multiple factors, including the environment of care (lack of necessary medications and fractured supply chain), insufficient and poorly trained healthcare providers, and lack of systems technology that supports adherence to best practices.

Notably lacking even in advanced health care systems are quality metrics that assess performance on diagnosis and management of common acute illnesses in the ambulatory care setting [5]. Most ambulatory care metrics focus on chronic disease management, leaving the performance on routine acute illnesses such as the diagnosis and management of urinary tract infection (UTI) largely unknown. UTI is a common diagnosis both worldwide and in Kenya, and is the most common reason for outpatient antibiotic prescription in Kenya [6]. The routine use of a guideline-recommended antibiotic has the potential to improve outcomes for patients with UTI as well as reduce antimicrobial resistance in local communities [6, 7].

Various methods of improving provider adherence to guidelines have been attempted, both in LMIC’s as well as highly resourced health systems. Educational interventions targeted at non-physician health care providers have shown variable levels of success depending on the circumstances of implementation [8]. In this present study we examine the influence of a series of brief educational interventions aimed at improving clinician guideline adherence for community acquired urinary tract infection in women aged 14–49. We hypothesize that a series of brief educational interventions will improve clinician adherence to guidelines and improve appropriate prescribing behavior. The primary outcome is the proportion of cases in which a clinician prescribed antibiotic therapy according to the practice guideline. The secondary outcome assessed was appropriate documentation of key elements of the history and physical exam relevant to the diagnosis.

## Methods

### Study sites

Two semi-urban primary health centers located in Kenya were selected for measurement of quality metrics and implementation of the brief educational interventions. Penda Health (PH) is a privately run chain of health centers targeting low-income Kenyans that sees approximately 1500 patients per month at its Kitengela site. AIC-Kijabe Hospital (AICKH) is a faith-based health center that sees approximately 2200 patients per month at its Naivasha site. Patients pay out of pocket for care at both sites. Care is administered by Kenyan clinical officers (CO’s)–non-physician health care providers who have completed a three-year diploma program and one-year supervised internship and who are licensed to provide comprehensive primary health care in Kenya.

Non-governmental health care services delivered by non-physician health care providers represent the bulk of primary care services delivered in Kenya [9]. Accordingly, these sites represent the best “real-world” environment for quality improvement in the Kenyan primary care setting. We sought to broaden the generalizability of our results by selecting both a private as well as a faith-based organization.

### Guideline development

A multidisciplinary panel of local experts, including Family Medicine and Internal Medicine reviewed and prioritized recommendations extracted from evidence-based national and international guidelines for the treatment of UTI. The panel reviewed the Kenyan Ministry of Health guidelines [7] and the medical literature [10, 11] and proposed indicators of quality of care for the screening, diagnosis, and treatment of this common condition. Content validity was assessed and reviewed over three rounds of meetings. Using an accepted framework regarding quality metric development [12], a set of critical history and physical findings to be documented were selected, consistent with international standards of practice and norms.

The key quality metrics assessed were the documentation of vaginal discharge, the patient’s pregnancy status, complete documentation of vital signs, the presence or absence of costovertebral angle tenderness, and the prescription of the guideline-recommended antibiotic or documentation or why an alternative was chosen [7, 13].

### Study intervention

At both sites, a series of brief educational interventions, each lasting less than one hour, were administered to clinical officers during protected training time on an average of four months apart (Appendix 1). The interventions were chosen based on our review of the available literature on how best to improve provider adherence to guidelines. While no single method is clearly superior, it seems that a combination of active approaches is better than passive dissemination of guidelines [14], and the use of local antibiotic resistance data has shown promise in at least one study [15]. We also sought to use interventions that are simple, replicable, and logistically feasible in resource-limited settings.

The first intervention involved introduction of the clinical guideline for management of uncomplicated urinary tract infection. This included a powerpoint presentation of the guideline led by a physician affiliated with the study. There was opportunity for discussion of the rationale behind the guideline and a question and answer session.

The second intervention involved CO’s reviewing each other’s documentation of patients with UTI as they pertain to the guideline in a peer-to-peer format. CO’s worked in pairs to review a UTI chart of their peers and were asked to provide feedback on whether the chart was in compliance with the clinical guideline and to suggest steps that would improve compliance. There was opportunity for discussion as a large group at the end of the session.

The third intervention involved the discussion of a recently published peer-reviewed Kenyan study describing local antimicrobial resistance patterns of uropathogens at a well known and respected private hospital [16]. This study reinforced the efficacy and rationale for the guideline-recommended antibiotic.

### Data collection

Data were collected at baseline and at approximately six-month intervals between educational interventions at both sites. The study authors collected a random sample of medical records of female patients ages 14 to 49 with a final diagnosis of UTI from each pre- and post-intervention period. Charts were scored according to the clinical practice guideline (Appendix 2). The key quality metrics assessed were the documentation of vaginal discharge, the patient’s pregnancy status, complete documentation of vital signs, the presence or absence of costovertebral angle tenderness, and the prescription of the guideline-recommended antibiotic or documentation of why an alternative was chosen. Where there was any question regarding a chart’s concordance with the guideline, reviewers consulted with each other to make a final determination. Ethical approval was granted by the AICKH IRB for confidential abstraction of clinical data without individual patient consent.

### Statistical analysis

Data were entered into Microsoft Excel (Microsoft Corp. Seattle, WA) with process measures scored as a binary variable (yes/no). Composite quality metric scores were calculated as a sum of successfully completed quality metrics, ranging from zero to five, with a maximum possible score of five. The primary outcome measure was the proportion of charts in compliance with the guideline on appropriate antibiotic therapy (Quality Metric #5, Appendix 2). Significance of a change in proportion was assessed with chi-squared tests. Composite quality scores were compared using unpaired two-tailed T-test with significance at p < 0.05. Logistic regression was used to determine significance of multiple possible confounders, including individual quality metrics, clinical site, patient age, provider, and intervention stage.

## Results

All clinical officers (N = 24), representing two different outpatient care clinic sites (11 from AIC Kijabe and 13 from Penda Health) received a short educational intervention. Chart review consisted of a total of 474 charts with a recorded diagnosis of UTI. All charts were scored according to each of the five key findings described as quality metrics.

**Fig 1:**
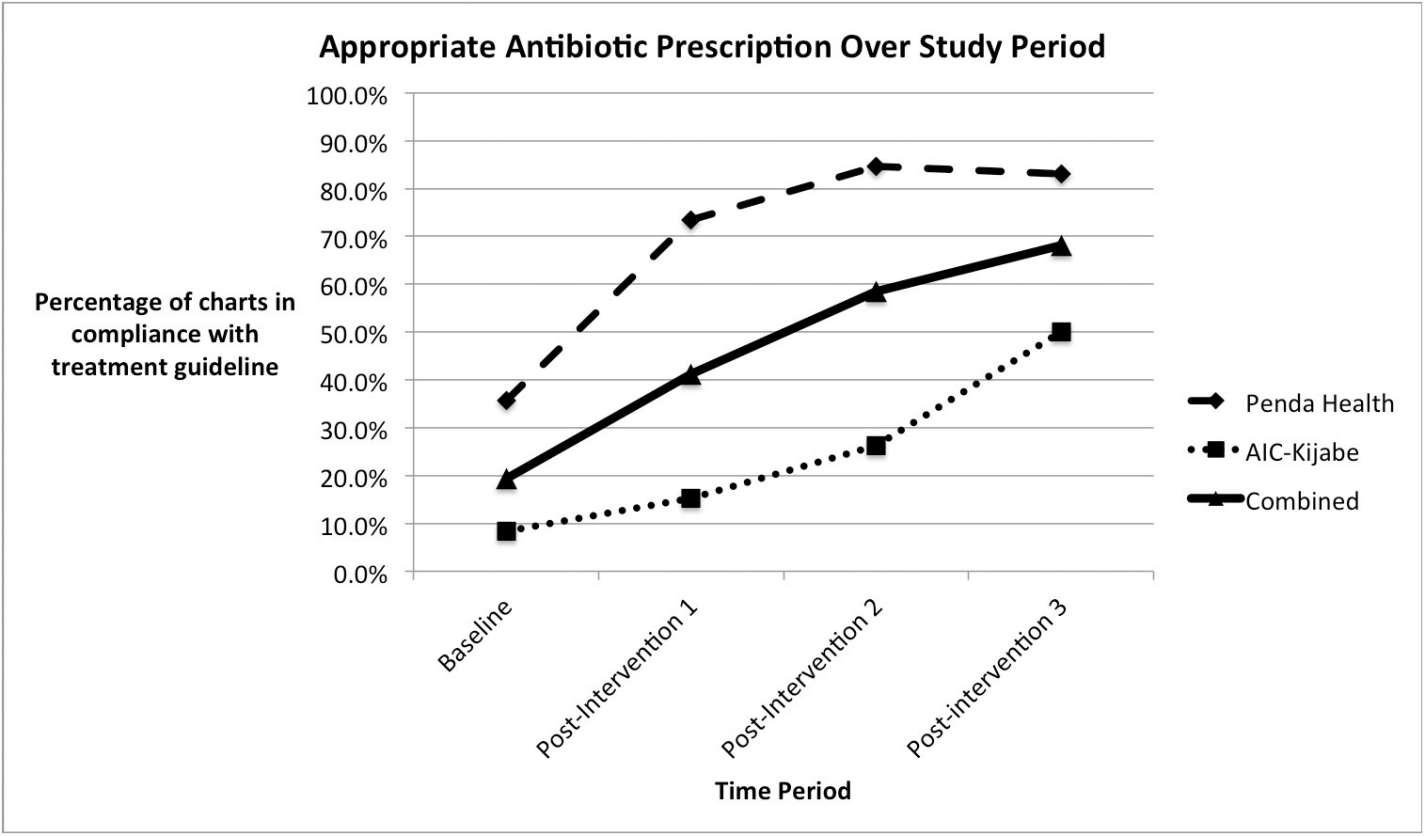
Percentage of charts in compliance with the primary quality metric, appropriate antibiotic prescription, during the study period. N = 474.

**Fig 2:**
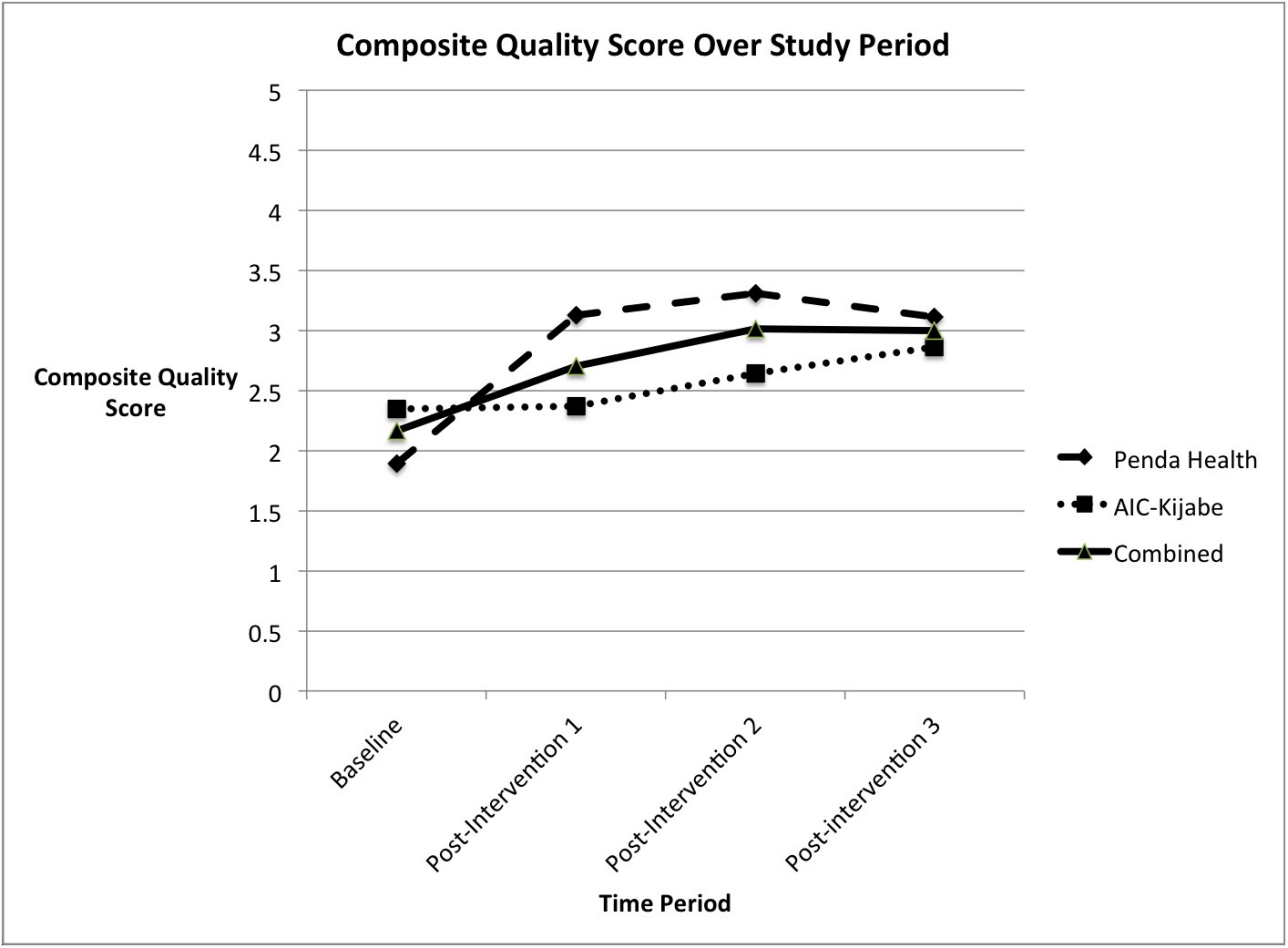
Composite Quality Score over the study period at both intervention sites. The score reflects quality metric compliance out of a maximum possible of five. N = 474.

Baseline performance on the primary outcome of appropriate antibiotic prescription was 19%, which had improved to 68% at the end of the study period (Χ^2^ = 150.7 [1 degree of freedom], p < 0.001) (Fig 1). The secondary outcome of composite quality score also improved significantly from an average of 2.16 to 3.00 on a five-point scale (unpaired T-test = 6.58, [degrees of freedom = 235], p < 0.0001) (Fig 2).

Interventions had different effects at different clinical sites. At Penda Health, baseline performance on the primary outcome of appropriate antibiotic prescription was 35.7%, which had improved to 83.0% at the end of the study period (Χ^2^ = 51.8 [1 degree of freedom], p < 0.001). At AICKH, initial appropriate antibiotic prescription was 8.3%, which improved to 50% at the end of the study period (Χ^2^ = 100.6, p < 0.001).

We performed logistic regression analysis to assess for possible confounders that may have contributed to this outcome (S3 Fig). Of the features we assessed, only intervention stage and clinical site were independent predictors of the primary outcome (p < 0.0001). Provider, patient age, and performance on other quality metrics were not significant at a conservative threshold of p < 0.05.

The secondary outcome measure, composite quality score, was 1.89 out of 5.0 at Penda Health at the beginning of the study period, and had improved to 3.11 following the educational interventions (unpaired T-test = 6.38, p < 0.001). At AICKH, the initial composite quality score was 2.34, and improved to 2.86 at the end of the study period (unpaired T-test = 3.09, p = 0.0024).

**Fig 3:**
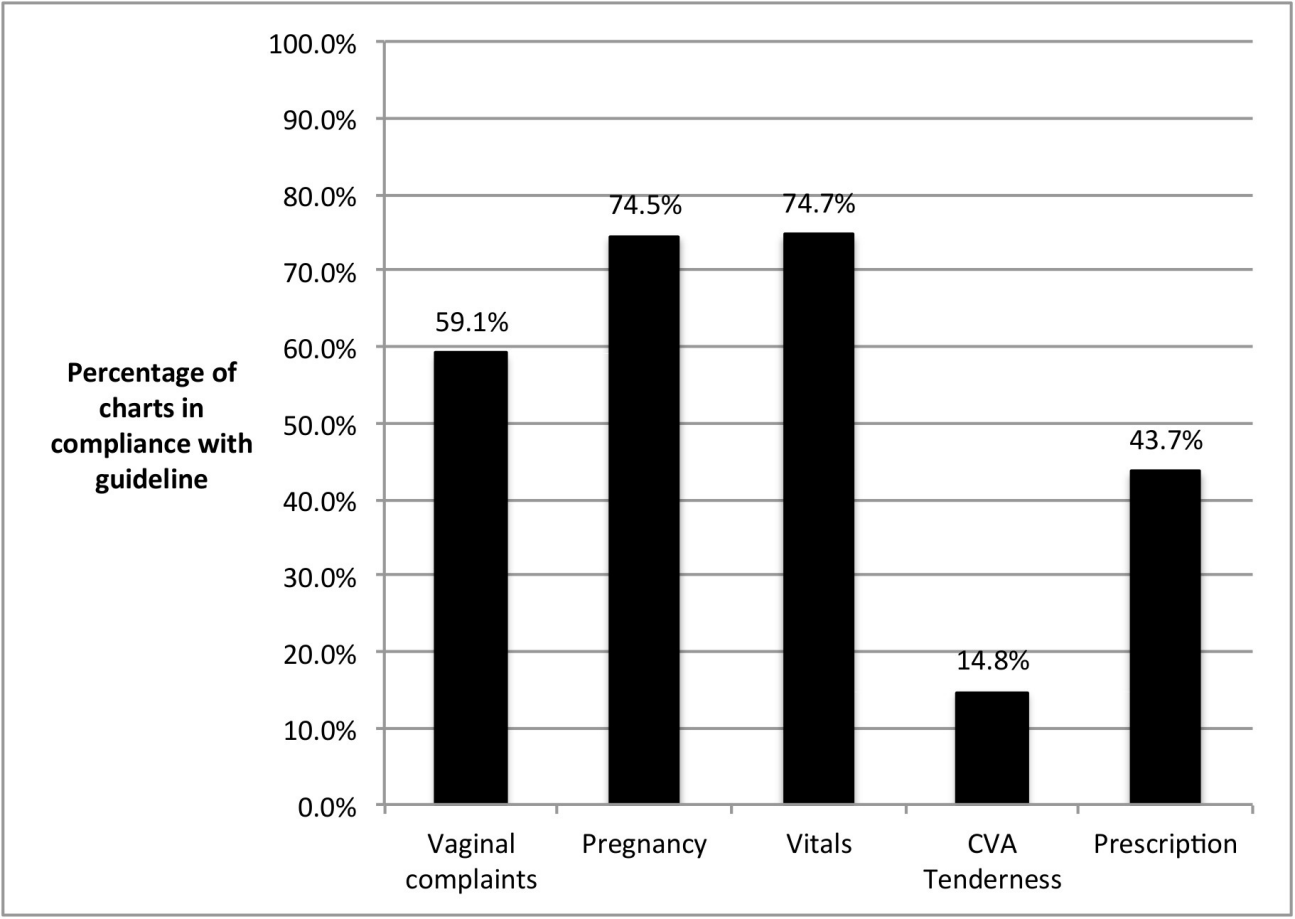
Individual quality metric compliance scores for both sites over the entire study period.

Overall performance on the five quality metrics across both sites over the entire study period revealed the highest performance on assessing pregnancy status and vital sign documentation (74.5% and 74.7% respectively). Documentation of costovertebral angle (CVA) tenderness was the least-performed metric, occurring in only 14.8% of all charts reviewed (Fig 3).

## Discussion

In this study we report the successful use of an educational programme aimed at improving clinician guideline adherence for a common acute illness, namely community acquired urinary tract infection in women aged 14–49. Our baseline results are consistent with prior studies of ambulatory care quality, showing overall poor adherence to clinical practice guidelines. Providers at both sites documented appropriate use of antibiotics in fewer than 40% of cases at baseline. An improvement in the primary outcome was noted in the period following each of the educational sessions at both study sites, except for the final intervention at PH, where adherence rates appeared to plateau at approximately 80%. Logistic regression confirmed that no other factors had a significant effect on the primary outcome.

The secondary outcome, composite quality scores, showed modest improvement from an average at both sites of 2.16 to an average of 3.00 at the end of the study period. This improvement was driven largely by the primary endpoint of antibiotic prescription, which improved significantly at both sites over the study period. Provider performance on quality metric #4 (CVA tenderness) was very poor throughout the study period (Fig 3). Through informal discussions with providers following completion of the study, it became clear that most providers did not recall how to perform this physical exam maneuver. We were able to easily correct this deficiency after the study period through a brief training on physical exam maneuvers. We wish to highlight that the measurement of quality metrics allowed us to identify a deficiency that was easily correctable through further training.

We theorized, building on prior research in this field, that a multi-faceted approach to quality improvement, as opposed to a single session, would be most effective in changing provider behavior. Our results show continual quality improvement over the study period, and suggest a “booster” effect from each intervention, all of which were completed within nine months at each site. It seems likely that if the study period were extended with further interventions targeted to particular deficiencies, such as training in CVA tenderness, further improvements would be realized. Our study was not designed to determine if any one intervention had greater effects than others, but our data suggest that some interventions may have had different magnitude of effects on quality metrics depending on the study site. Further research is needed to identify educational methodologies that lead to the greatest sustained behavior change among healthcare providers, as well as population level studies to determine cost-benefit analysis and when to utilize urine culture, an expensive, yet useful diagnostic test.

Gathara *et al* have shown that there is significant variability in quality of care among hospitals, and that inter-hospital variability may in fact surpass inter-clinician variability [17]. They hypothesize that organizational attributes may have more impact on care quality than the effort or skill of individual providers. This may explain the finding in our study that PH achieved a plateau of 80% on the primary outcome far earlier than AICKH. We believe that differences in provider turnover, a robust provider onboarding process (including orientation to clinical guidelines), and regular feedback from supervisors at PH may have contributed to these differences.

Our study has several limitations. First, we rely on retrospective chart review to assess process measures performed in a clinical encounter. Thus, underestimation of quality performance may occur in the setting of inadequate documentation (i.e. a provider truly did assess vaginal symptoms, but failed to document it), and overestimation is also possible (i.e. a provider may have falsely documented a physical exam maneuver in order to appear more thorough). Mwaniki and colleagues recently compared retrospectively collected quality metrics with prospectively collected data and found high concordance within Kenyan hospitals, validating our use of retrospectively collected quality data [2]. Furthermore, since an antibiotic was prescribed in the vast majority of cases for this condition, we believe that omission or documentation bias is unlikely to have affected our primary outcome.

Second, the quality metrics themselves reflect process measures as opposed to clinical outcomes. While the metrics were chosen with a strong clinical rationale in mind, it must be noted that the link between each process and clinical outcomes has not yet been established. However, the appropriate use of antibiotics for UTI has been studied extensively elsewhere, and improved microbiological and clinical cure rates when uropathogens are sensitive to first-line antibiotics has been demonstrated [13]. Thus, when considered with local antimicrobial resistance patterns, this process measure is likely highly correlated with clinical outcome. Furthermore, the alarming rates of gram-negative resistance to broad-spectrum antibiotics in Kenya make the judicious use of antimicrobial therapy for UTI critical for public health reasons [6].

Finally, our study was conducted at only two outpatient sites in Kenya, and thus may have limited generalizability to other facilities. We sought to limit this bias by conducting the study in two different settings–a for-profit chain of urgent care centers, and a faith-based non-profit organization. Although the majority of primary health care is provided by the private sector in LMICs (including NGOs), it would be interesting to see the effects of these brief educational interventions in government-run health centers.

## Conclusions

The need for robust quality improvement programs in LMICs is well known. While much work has been done globally to measure and improve the quality of care in the inpatient setting, measurement of quality for acute illnesses in the ambulatory care setting lags far behind. In this study, we have shown a series of brief educational interventions that were associated with significant improvement on appropriate antibiotic prescribing for urinary tract infections. This area is ripe for further study, with opportunities to expand quality metrics to a broader range of conditions, to refine educational interventions to make them more effective, and to expand the settings in which these data are collected. It is also very important that we validate process measures by linking them to clinical outcomes. The measurement and continuous improvement of quality of care in the ambulatory setting is critical to improving health in vulnerable populations.

## Supporting information

S1 Fig Timeline of Educational Interventions. (PDF)

S2 Fig Quality Metrics. (PDF)

S3 Fig Logistic Regression–Table of Coefficients. (PDF)

S1 Table UTI Data Set Coded for Logistic Regression (CSV). (CSV)

S2 Table Raw Data Set. (XLSX)
