# Supplementary material for: Brief educational interventions to improve performance on novel quality metrics in ambulatory settings in Kenya: A multi-site pre-post effectiveness trial
Source: PLoS One. 2017 Apr 14;12(4):e0174566. doi: 10.1371/journal.pone.0174566 (PMC5391918; doi:10.1371/journal.pone.0174566)
Supplement: S1 Fig — (PDF) [file pone.0174566.s001.pdf]

S1 Fig: Timeline of Educational Interventions

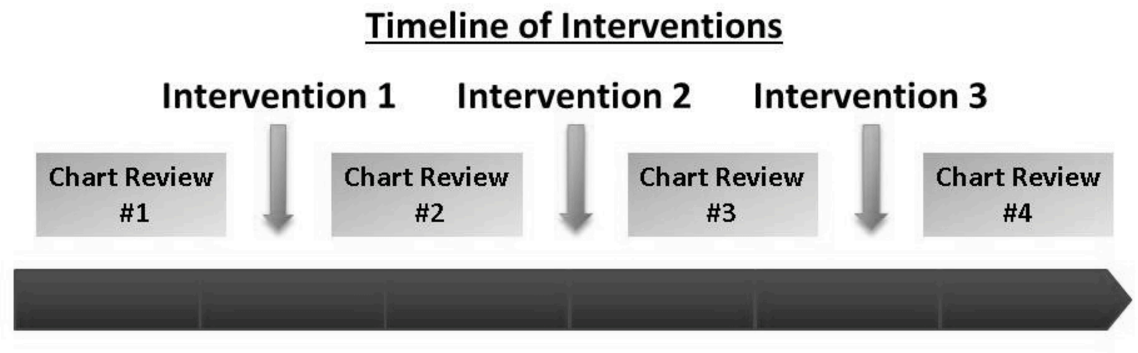

Intervention 1: Introduction of clinical guideline with PowerPoint presentation and discussion.

Intervention 2: Clinical officers review UTI charts of their peers and offer suggestions for how adherence to clinical practice guideline could be improved.

Intervention 3: Discussion of peer-reviewed literature describing local antibiotic resistance data [15].
