# Supplementary material for: Brief educational interventions to improve performance on novel quality metrics in ambulatory settings in Kenya: A multi-site pre-post effectiveness trial
Source: PLoS One. 2017 Apr 14;12(4):e0174566. doi: 10.1371/journal.pone.0174566 (PMC5391918; doi:10.1371/journal.pone.0174566)
Supplement: S2 Fig — (PDF) [file pone.0174566.s002.pdf]

S2 Fig: Quality Metrics for Urinary Tract Infection

Quality Metric	Measurement	Rationale
**Assess vaginal complaints**	Documentation of presence/absence of vaginal discharge or itching	The presence of vaginal discharge may lead the clinician down an alternative diagnostic pathway
**Assess Pregnancy Status**	Documentation of any of the following:<br>• Highly-effective contraceptive method (IUD or Implant)<br>• LMP within the last one month<br>• Urine pregnancy test results<br>• Known pregnancy<br>• Active breastfeeding or recent childbirth<br>• No intercourse since most recent menstruation	Pregnancy status helps guide the clinician in correct antibiotic prescription and is an important consideration in the management of UTI.
**Complete Vital Signs**	Temperature, heart rate, and blood pressure are recorded	Vital sign abnormalities alert the clinician to the severity of infection and possible upper-tract infection (pyelonephritis)
**Costovertebral Angle (CVA) Tenderness**	Under the physical exam section, the presence or absence of CVA tenderness is documented (flank, loin, or renal angle tenderness are acceptable alternatives)	CVA tenderness suggests pyelonephritis, which would mandate the use of an alternative therapy to the guideline-recommended antibiotic, nitrofurantoin.
**Appropriate antibiotic prescription**	Under the treatment section, there is documentation of the guideline recommended antibiotic (Nitrofurantoin 100 mg bid for 5-7 days) – OR – a reason why an alternative was chosen (e.g. ciprofloxacin for pyelonephritis, TMP-SMX due to previous GI intolerance with nitrofurantoin, or nausea in pregnancy, etc.)	Nitrofurantoin has the most favorable resistance profile in Kenya [15] and is well-tolerated and readily available at a low cost.
