# Supplementary material for: Brief educational interventions to improve performance on novel quality metrics in ambulatory settings in Kenya: A multi-site pre-post effectiveness trial
Source: PLoS One. 2017 Apr 14;12(4):e0174566. doi: 10.1371/journal.pone.0174566 (PMC5391918; doi:10.1371/journal.pone.0174566)
Supplement: S3 Fig — (PDF) [file pone.0174566.s003.pdf]

S3 Fig: Table of Coefficients for Logistic Regression

Variable	Co-efficient	p-value
Intervention	0.7042	< 0 .0001
Site	-3.0913	< 0.0001
Provider	0.0791	0.1029
Patient Age	-0.0197	0.2093
Vaginal Discharge Documentation	-0.0533	0.8293
Pregnancy Status Documentation	0.0455	0.8665
Vital Sign Documentation	-0.1957	0.4999
CVA Tenderness Documentation	0.2919	0.3819

This table shows the coefficient of effect of independent variables on the dependent variable of appropriate antibiotic prescription.
